# 4-Cl-edaravone and (*E*)-2-chloro-3-[(*E*)-phenyldiazenyl]-2-butenoic acid are the specific reaction products of edaravone with hypochlorite

**DOI:** 10.3164/jcbn.19-115

**Published:** 2020-04-09

**Authors:** Sakiko Amekura, Misuzu Nakajima, Mami Watanabe, Makoto Saitoh, Sayaka Iida, Yorihiro Yamamoto, Akio Fujisawa

**Affiliations:** 1 School of Bioscience and Biotechnology, Tokyo University of Technology, 1404-1 Katakura-cho, Hachioji, Tokyo 192-0982, Japan

**Keywords:** edaravone, hypochlorite, inflammation, two-electron oxidants, two-electron reductants

## Abstract

3-Methyl-1-phenyl-2-pyrazolin-5-one (edaravone) is a synthetic one-electron antioxidant used as a drug for treatment against acute phase cerebral infarction in Japan. This drug also reacts with two-electron oxidants like peroxynitrite to give predominantly 4-nitrosoedaravone but no one-electron oxidation products. It is believed that this plays a significant role in amelioration of amyotrophic lateral sclerosis. The drug was approved for treatment of amyotrophic lateral sclerosis in Japan and USA in 2015 and 2017, respectively. In this study, we examined the reaction of edaravone with another two-electron oxidant, hypochlorite anion (ClO^−^). Edaravone reacted with ClO^−^ in 50% methanolic phosphate buffer (pH 7.4) solution containing typical two-electron reductants, such as glutathione, cysteine, methionine, and uric acid, as internal references. The concentration of edaravone decreased at a similar rate as each co-existing reference, indicating that it showed comparable reactivity toward ClO^−^ as those references. Furthermore, 4-Cl-edaravone and (*E*)-2-chloro-3-[(*E*)-phenyldiazenyl]-2-butenoic acid (CPB) were identified as primary and end products, respectively, and no one-electron oxidation products were detected. These results suggest that edaravone treatment can bring greater benefit against ClO^−^-related injury such as inflammation, and 4-Cl-edaravone and CPB can be good biomarkers for ClO^−^-induced oxidative stress.

## Introduction

**Fig. 1:**
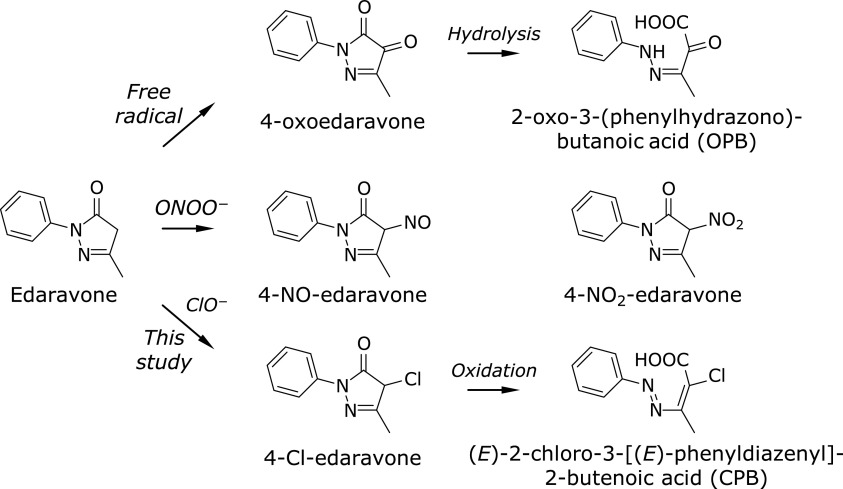
Edaravone and its oxidation products induced by free radical, peroxynitrite (ONOO^−^), and hypochlorite (ClO^−^, this study).

3-Methyl-1-phenyl-2-pyrazolin-5-one (edaravone, Fig. 1) is a synthetic one-electron antioxidant that has been applied for treatment against acute phase cerebral infarction since 2001 in Japan. We demonstrated that edaravone strongly inhibited the oxidation of soy-phosphatidylcholine liposomal membrane induced by a lipid-soluble radical initiator 2,2'-azobis(2,4-dimethylvaleronitrile) (AMVN) or a water-soluble 2,2'-azobis(2-amidinopropane) dihydrochloride (AAPH).^(1)^ The one-electron oxidation products were 3-methyl-1-phenyl-2-pyrazolin-4,5-dione (4-oxoedaravone) and its hydrolyzate, 2-oxo-3-(phenylhydrazono)butanoic acid (OPB).^(1)^ Kawai *et al.*^(2)^ also detected OPB in the ischemic brains of rats.

Edaravone also reacted with two-electron oxidants like peroxynitrite, yielding predominantly 3-methyl-4-nitroso-1-phenyl-2-pyrazoline (4-NO-edaravone), while one-electron oxidation products like 4-oxoedaravone and OPB were absent.^(3)^ Edaravone was approved for treatment of amyotrophic lateral sclerosis (ALS) in 2015 in Japan and in 2017 in USA, and peroxynitrite was suggested to play an important role in ALS.^(4)^ We showed that edaravone could react with ONOO^−^ 30-fold faster than uric acid (UA).^(3)^ The second-order rate constant for reaction of UA and ONOO^−^ has been estimated as 1.5 × 10^2^ or 4.8 × 10^2^ M^−1^s^−1^.^(5,6)^ Considering that second-order rate constants of tyrosine and tryptophan were also estimated as 5.5 × 10^3^ and 1.84 × 10^2^ M^−1^s^−1^, respectively,^(7,8)^ the reactivity of edaravone toward ONOO^−^ is high.

In this study, we examined another biologically important two-electron oxidant, hypochlorite anion (ClO^−^), because its specific oxidation products have not been characterized. Biologically, ClO^−^ is formed from a reaction of Cl^−^ and hydrogen peroxide (H_2_O_2_) catalyzed by myeloperoxidase (MPO), which is released from activated neutrophils during inflammation. It is a strong reactive oxygen species (ROS) and induces characteristic oxidation reactions such as sulfide oxidation to form sulfoxide and H_2_O_2_ oxidation to produce singlet oxygen (^1^O_2_). Since ClO^−^ shows a strong bactericidal effect against germs including bacteria and Norwalk virus, it plays an important role in immune action. However, if ClO^−^ is produced excessively, it can cause oxidative damage to tissues.

In this study, the reactivity of edaravone toward ClO^−^ and its reaction products were investigated. We estimated the reactivity by comparing it with two-electron reductants such as reduced glutathione (GSH), cysteine (Cys), methionine (Met), and UA. Edaravone and each reference co-existed in a homogeneous solution and were oxidized with sodium hypochlorite (NaClO) induced at a constant rate into the solution. The amount of edaravone decreased a little slower than GSH or Cys did, almost identical with Met, and faster than UA, during competitive reactions with these antioxidants. These results suggested that edaravone has comparable reactivity to those antioxidants. Furthermore, two reaction products were identified. 4-Cl-edaravone was formed as a primary oxidation product by edaravone oxidation, which was then further oxidized to (*E*)-2-chloro-3-[(*E*)-phenyldiazenyl]-2-butenoic acid (CPB) (Fig. 1). From these results, edaravone treatment against ClO^−^-related injury such as inflammation may be useful and 4-Cl-edaravone can be a good marker to indicate ClO^−^ formation *in vivo*.

## Materials and Methods

### Chemicals

Edaravone, GSH, Cys, Met, UA, NaClO aqueous solution, and other chemicals were purchased from FUJIFILM Wako Pure Chemical Industries, Ltd. (Osaka, Japan). Methanolic solutions of edaravone were prepared and stored at −20°C until use.

### Reaction of edaravone or 4-Cl-edaravone with ClO^−^

Edaravone was dissolved in 50% methanolic phosphate buffer solution (40 mM, pH 7.0) containing 100 µM diethylenetriaminepentaacetic acid (DTPA) as a chelator with GSH, Cys, Met, or UA as an internal reference. The final concentrations of edaravone and the internal reference were approximately 25 µM. NaClO solution (16.1 mM) was induced into the well-stirred reaction mixture (30 ml) at a constant rate (0.5 µl/min) using a syringe pump (Harvard Apparatus, Holliston, Massachusetts). Changes in concentrations of edaravone and the internal reference were measured every 25 or 30 min using an optimized HPLC as described below.

Oxidation of edaravone or 4-Cl-edaravone alone was also conducted. Edaravone and 4-Cl-edaravone solutions (50% methanolic or 100% methanolic) were prepared. NaClO solution was added instantaneously or introduced gradually (1.0 µM/min) to the edaravone solution. Changes in concentrations of edaravone and its metabolites were determined every 30 min during reaction using the HPLC system.

### Isolation of 4-Cl-edaravone and CPB

For identification of reaction products, 4-Cl-edaravone and CPB were isolated. After 30 min reaction of edaravone (1.0 mM) and excess NaClO, the reaction mixture was analyzed by HPLC to confirm that edaravone was completely reacted. The solution containing 4-Cl-edaravone and CPB was loaded onto a C18 solid phase extraction (SPE) cartridge (DSC-18, 2 g/12 ml, Sigma-Aldrich Japan, Tokyo, Japan) and flushed out to separate 4-Cl-edaravone adsorbed to the SPE cartridge. After washing the SPE cartridge with sufficient pure water, 4-Cl-edaravone was eluted with methanol. Next, a small amount of diluted HCl was added to the flow through, and the solution was loaded onto another C18 SPE cartridge. After washing with diluted HCl, CPB was eluted with methanol. After checking the purities, both solvents were removed by a rotary evaporator, and stored at −20°C until use.

The MS spectra of the collected 4-Cl-edaravone and CPB were measured by using a HPLC equipped with a time-of-flight mass spectrometer (LC/TOFMS).

### HPLC analysis

The changes in concentrations of edaravone and the internal references were determined by a reverse phase HPLC equipped with a UV detector monitoring absorption at 210 nm (for Met analysis) or 280 nm (for UA analysis) and an electrochemical detector (ECD) connected in tandem (for edaravone, GSH, and Cys analysis). The applied voltage of the electrochemical detector against an Ag/AgCl reference electrode was 600 mV. A 25% methanol aqueous solution containing NaClO_4_ (100 mM) and phosphate (0.1%) was delivered at 1.0 ml/min as a mobile phase. The separation column was a TSKgel ODS-100Z (5 µm, 150 mm × 4.6 mm; TOSOH, Tokyo, Japan). To analyze 4-Cl-edaravone and CPB, which are oxidation products of edaravone and ClO^−^, another HPLC was used. The mobile phase and separation column were 40% methanol aqueous solution containing 24 mM NaH_2_PO_4_ delivered at 1.0 ml/min and a Wakopak Navi C18-5 (5 µm, 250 mm × 4.6 mm; FUJIFILM Wako Pure Chemical Corporation, Osaka, Japan), respectively. Detection was carried out by monitoring the absorbance at 240 nm.

### LC/TOFMS analysis

To obtain accurate mass-to-charge ratios (*m/z*) of edaravone oxidative metabolites, a HPLC equipped with a TOF-MS (JMS-T100LC; JEOL Ltd., Tokyo, Japan) was used. Negative ionization was performed at an ionization potential of −2,000 V. For measuring parent molecular ions, the optimized applied voltages to the ring lens, outer orifice, inner orifice, and ion guide were −5 V, −20 V, −5 V, and −500 V, respectively. To obtain accurate *m/z* values, trifluoroacetic acid (TFA) was used as an internal standard for *m/z* calibration. To observe fragmentation, the above potential settings were changed to −10 V, −60 V, −10 V, and −500 V, respectively.

The HPLC conditions were as follows. 50% Methanol containing 0.1% formic acid was delivered at 1.0 ml/min as a mobile phase. A Wakopak Navi C18-5 (5 µm, 250 mm × 4.6 mm; FUJIFILM Wako Pure Chemical Corporation, Osaka, Japan) was used as a separation column. Approximately a quarter of the mobile phase was induced to the MS analyzer using a splitter.

## Results and Discussion

### Reactivity of edaravone toward ClO^−^

**Fig. 2:**
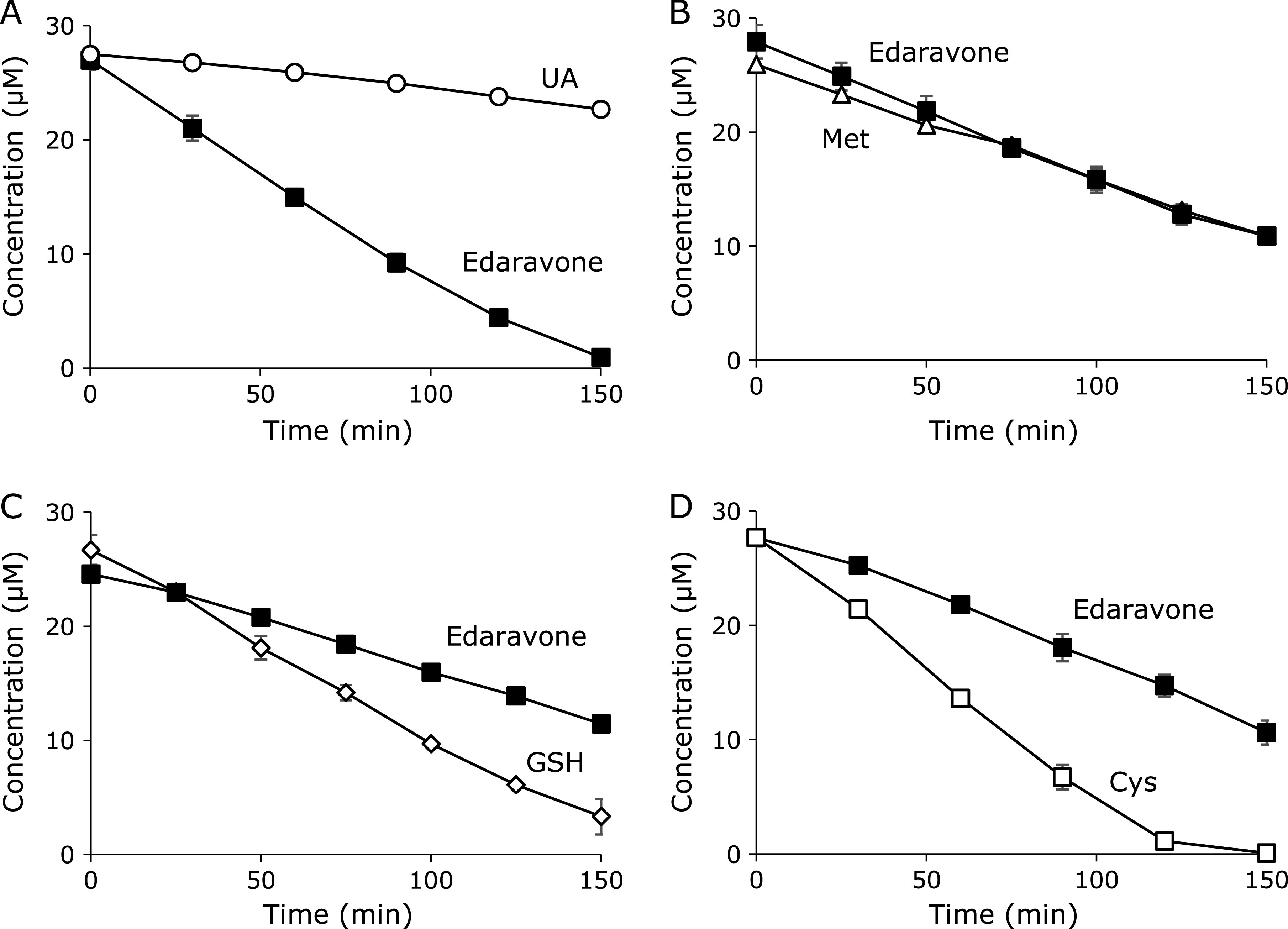
Time courses of changes in concentrations of edaravone and each antioxidant as an internal reference, during competitive reactions caused by a constant rate induction of NaClO. vs UA (A), vs Met (B), vs GSH (C), and vs Cys (D).

The reaction of edaravone and ClO^−^ was conducted in the presence of a two-electron reductant as an internal reference. Edaravone decreased at comparable rates with reference antioxidants such as UA, Met, GSH, and Cys (Fig. 2A–D). Edaravone and each reference antioxidant reacted with ClO^−^ and its concentration decreased competitively. The ratio of pseudo-second order rate constants (*k*_E_/*k*_R_) was given by equation 3 as described previously.^(3)^

$$Edaravone\;+\;{ClO}^{-}\overset{\;\;k_{E}\;\;}{\to }$$ (1)

$$Reference\;+\;{ClO}^{-}\overset{\;\;k_{R}\;\;}{\to }$$ (2)

$$k_{E}/k_{R}=\frac{\text{log}\left(\left[\text{Edaravone}\right]/{\left[\text{Edaravone}\right]}_{\text{0}}\right)}{\text{log}\left(\left[\text{Reference}\right]/{\left[\text{Reference}\right]}_{\text{0}}\right)}$$ (3)

Table 1 shows the *k*_E_*/k*_R_ values for edaravone vs each reference. The results suggest that the reactivity of edaravone toward ClO^−^ is comparable with the reference antioxidants. Second-order rate constants for the reactions of HOCl with Cys, GSH, and Met were determined by Storkey *et al.*^(9)^ to be 3.6 × 10^8^, 1.2 × 10^8^, and 3.4 × 10^7^ M^−1^s^−1^, respectively. The rate constant for UA was also estimated to be 3 × 10^5^ M^−1^s^−1^.^(10–12)^ Taken together, the pseudo second-order rate constant for reaction of edaravone and ClO^−^ can be estimated to be ~10^6^–10^7^ M^−1^s^−1^. Thiol (Cys) and sulfide (Met) residues are believed to be primary scavengers of ClO^−^ *in vivo* and their second-order rate constants for the reaction with ClO^−^ were estimated to be 3.0 × 10^7^ and 3.8 × 10^7^ M^−1^s^−1^, respectively.^(13)^ Edaravone was shown to possess enough reactivity to compete with these endogenous ClO^−^ scavengers.

### Identification of reaction products of edaravone and hypochlorite

**Fig. 3:**
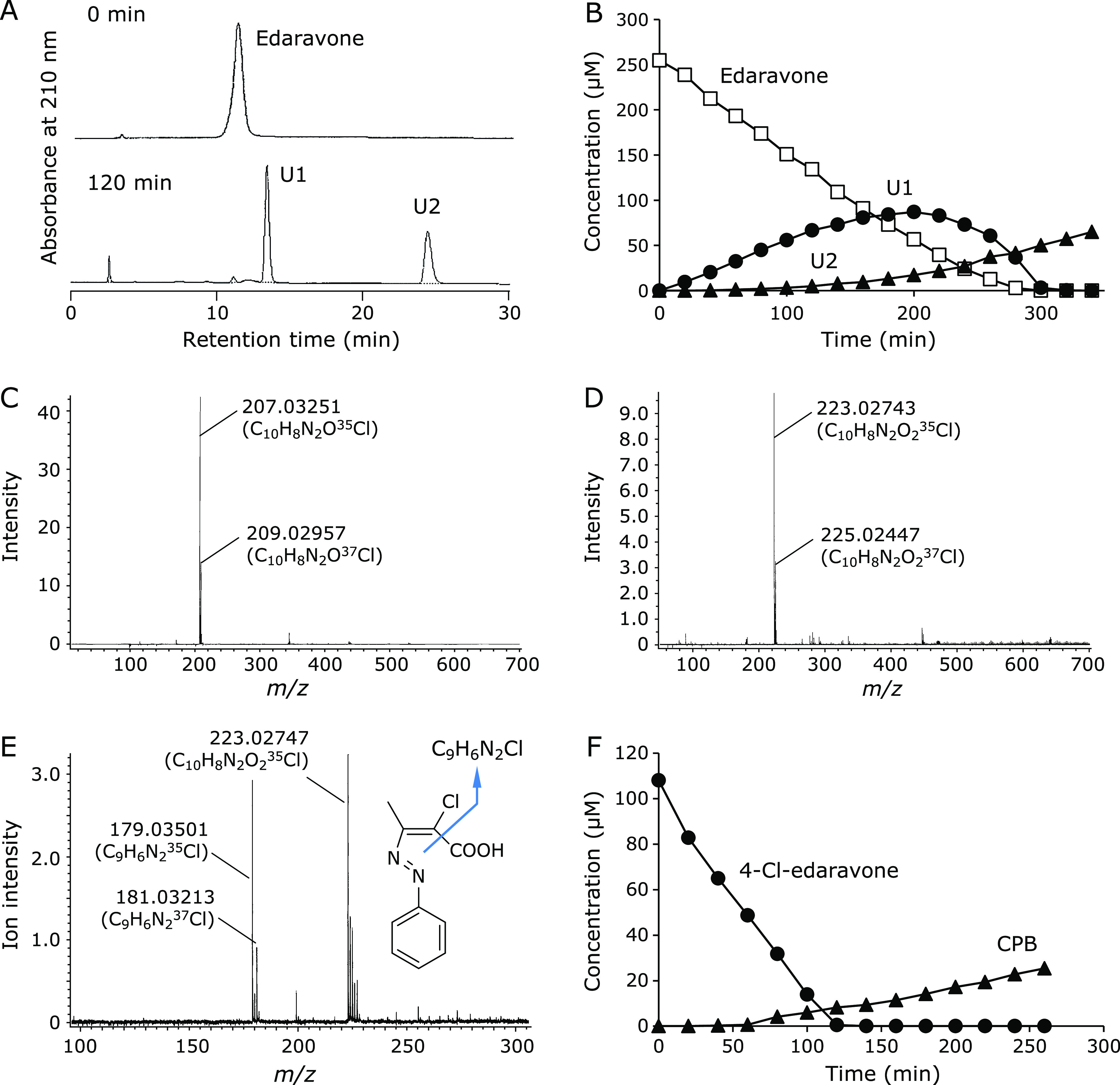
Formation of U1 and U2 during edaravone or 4-Cl-edaravone oxidation caused by NaClO induced at a constant rate and MS spectra of U1 and U2. (A) Chromatograms of reaction mixture at 0 min (upper) and 60 min induction (lower). (B) Time course of changes in concentrations of edaravone (□), U1 (●), and U2 (▲). MS spectra of U1 (C) and U2 (D). (E) Fragmentation of a purified CPB measured by LC/TOFMS. Spectra were measured by an optimized LC/TOFMS and their *m/z* values were compensated with TFA. (F) CPB (▲) formation during 4-Cl-edaravone (●) oxidation caused by a constant rate induction of NaClO at pH 7.0.

To identify the reaction products of edaravone and ClO^−^, edaravone alone was oxidized by ClO^−^. The formation of two unknown products U1 and U2 was observed after the reaction (Fig. 3A). Fig. 3B shows time course of changes of edaravone, U1 and U2 during induction of NaClO solution at a constant rate. U1 was formed in accordance with the consumption of edaravone and began decreasing when edaravone was depleted, suggesting that U1 was a primary product. U2 was produced in the late stage of the reaction and increased even after edaravone disappeared, indicating that U2 was a secondary product. MS spectra of U1 and U2 were measured by LC/TOFMS with negative electrospray ionization (Fig. 3C and D, respectively). Their accurate mass-to-charge ratios (*m/z*) were determined to be –207.03251 and –223.02743, respectively, using TFA as an internal standard. The postulated chemical formulas (theoretical *m/z* values) of U1 and U2 were C_10_H_8_N_2_O^35^Cl (–207.03252) and C_10_H_8_N_2_O_2_^35^Cl (–223.02743). Both formulas were supported by their ^37^Cl derived monoisotopic ions: Found *m/z* –209.02957; Theoretical –209.02957 (U1), and Found –225.02447; Theoretical –225.02448 (U2). Thus, the chemical structures of U1 and U2 were speculated to be 4-Cl-edaravone and (*E*)-2-chloro-3-[(*E*)-phenyldiazenyl]-2-butenoic acid (CPB) (Fig. 1). The C4 carbon of the pyrazoline ring of edaravone shows reactivity to ROS and 4-adducts are formed as the oxidation products; 4-oxoedaravone by radical-induced oxidation^(1)^ and 4-NO- and 4-NO_2_-edaravone are formed by ONOO^−^^(3)^ (Fig. 1). From these results formation of 4-Cl-edaravone as the primary product is well plausible to reaction of edaravone and ClO^−^. In order to confirm whether U2 is CPB, its fragmentation was examined. Using the optimized TOFMS setting for measuring fragmentation described in Materials and Methods, fragmentation of CPB was observed (Fig. 3E). In addition to the parent ion (*m/z* –223.02747), a fragment ion (*m/z* –179.03501) was observed. The fragment ion was due to decarboxylation (C_9_H_6_N_2_^35^Cl, theoretical *m/z* –179.03492), which was supported by its monoisotopic ion (C_9_H_6_N_2_^37^Cl, Found *m/z* –181.03213; Theoretical *m/z* –181.03197). These results strongly suggest that U2 has a carboxyl group in its chemical structure as expected.

Since CPB was suggested to be the secondary product, further oxidation of 4-Cl-edaravone was conducted. Isolated and purified 4-Cl-edaravone was reacted with constantly induced NaClO. CPB was produced by 4-Cl-edaravone oxidation (Fig. 3F), indicating CPB was the oxidation product of 4-Cl-edaravone and ClO^−^. On the other hand, CPB was not oxidized further by ClO^−^ (data not shown), suggesting that CPB is a stable end product.

### Postulated mechanism for 4-Cl-edaravone and CPB formation

**Fig. 4:**
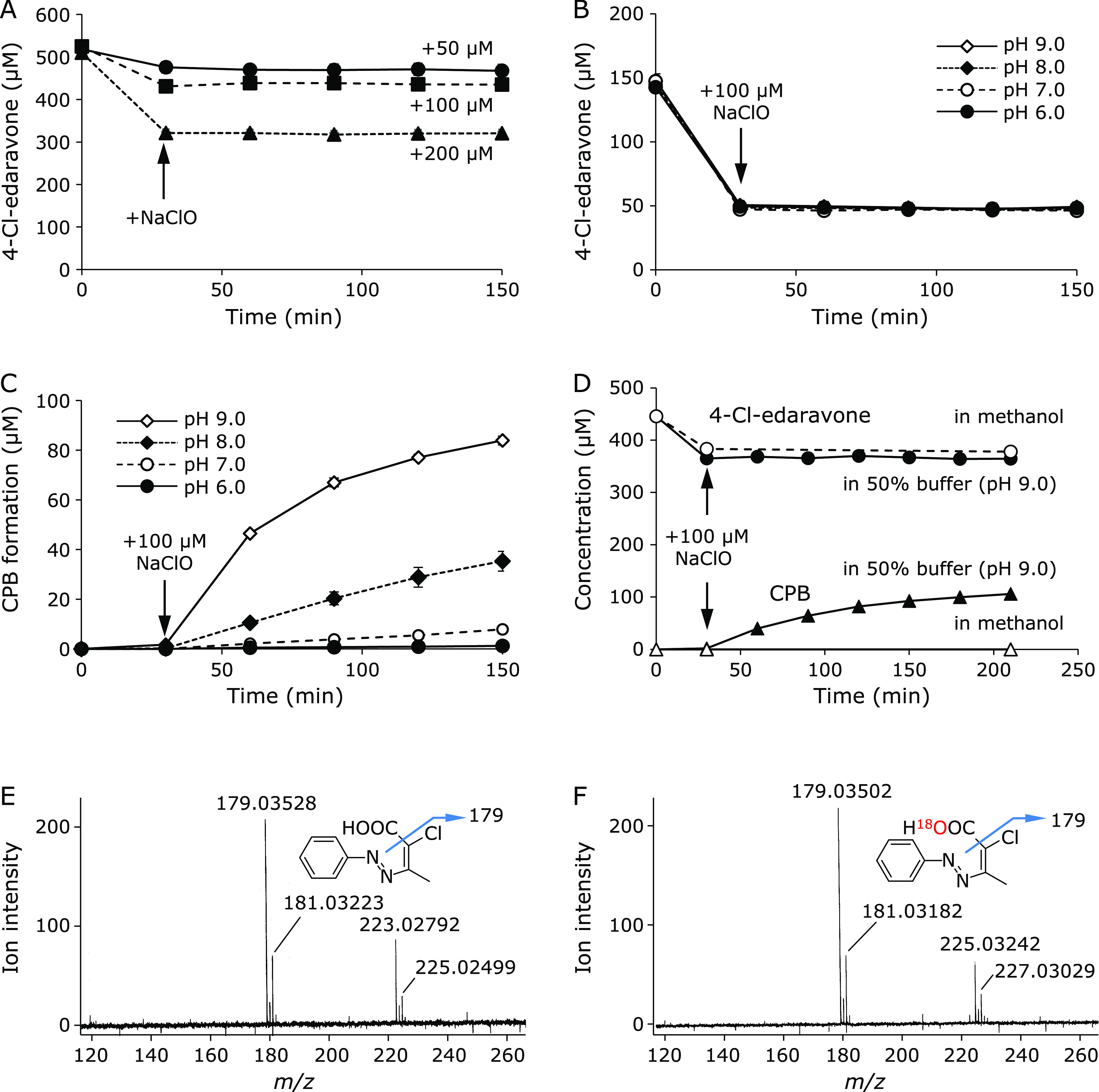
4-Cl-Edaravone oxidation by instantaneously added NaClO. (A) 4-Cl-Edaravone (500 µM) oxidation by 50 (●), 100 (■), or 200 µM (▲) NaClO addition at pH 7.0. (B) 4-Cl-Edaravone (150 µM) oxidation caused by 100 µM NaClO at pH 6.0 (●), 7.0 (◯), 8.0 (◆), and 9.0 (◇). (C) CPB formation after addition of 100 µM NaClO to 150 µM 4-Cl-edaravone at pH 6.0 (●), 7.0 (◯), 8.0 (◆), and 9.0 (◇). (D) 4-Cl-edaravone degradation (circles) and CPB (triangles) formation with an immediate reaction of 4-Cl-edaravone and NaClO in 50% buffer (pH 9.0) (closed symbols) or in methanol (opened symbols). MS spectra of CPB formed in non-isotopic water (E) and in H_2_^18^O (F) observed by optimized TOFMS. The *m/z* values of all ions were compensated by TFA. Inserted structures show plausible fragmentations.

In order to determine the formation mechanism for 4-Cl-edaravone and CPB, the stoichiometry for the reactions of edaravone and ClO^−^ and of 4-Cl-edaravone and ClO^−^ was investigated. The reaction of 4-Cl-edaravone and ClO^−^ was examined first because CPB is a non-reactive end product. 50, 100, and 200 µM of NaClO were added to 500 µM 4-Cl-edaravone solution at pH 7.0. The concentration of 4-Cl-edaravone immediately decreased after NaClO addition in accordance with the amount of added NaClO, with no further changes for 150 min (Fig. 4A). Next, 100 µM NaClO was added to 150 µM 4-Cl-edaravone solutions at pH 6.0, 7.0, 8.0, and 9.0. At all pHs, the concentration of 4-Cl-edaravone instantaneously decreased to 50 µM (Fig. 4B). A stoichiometric number expressed by the ratio of NaClO addition to 4-Cl-edaravone decrement was determined as approximately 1.0 for any concentration of NaClO and for any pH (Table 2). On the other hand, CPB formation was dependent on pH with the formation rates faster at higher pH (Fig. 4C). Furthermore, the concentration of 4-Cl-edaravone decreased by NaClO without CPB formation in 100% methanolic solution, whereas CPB was formed in 50% buffer (pH 9.0) (Fig. 4D). These results suggested that CPB formation requires H_2_O. Therefore, we conducted a reaction of 4-Cl-edaravone with NaClO in methanol/H_2_^18^O solution and the MS spectrum of CPB was collected. The MS spectrum of CPB formed in H_2_^18^O containing solution showed the parent ion (*m/z* –225.03242, Fig. 4F) two mass-units larger than that formed in non-isotopic H_2_O (*m/z* –223.02792, Fig. 4E), indicating that an oxygen atom of H_2_O was incorporated into the CPB molecule. Moreover, its decarboxylated fragment ion was non-isotopic (*m/z* –179.03502), suggesting that the oxygen atom derived from water was located on the carboxyl group.

**Fig. 5:**
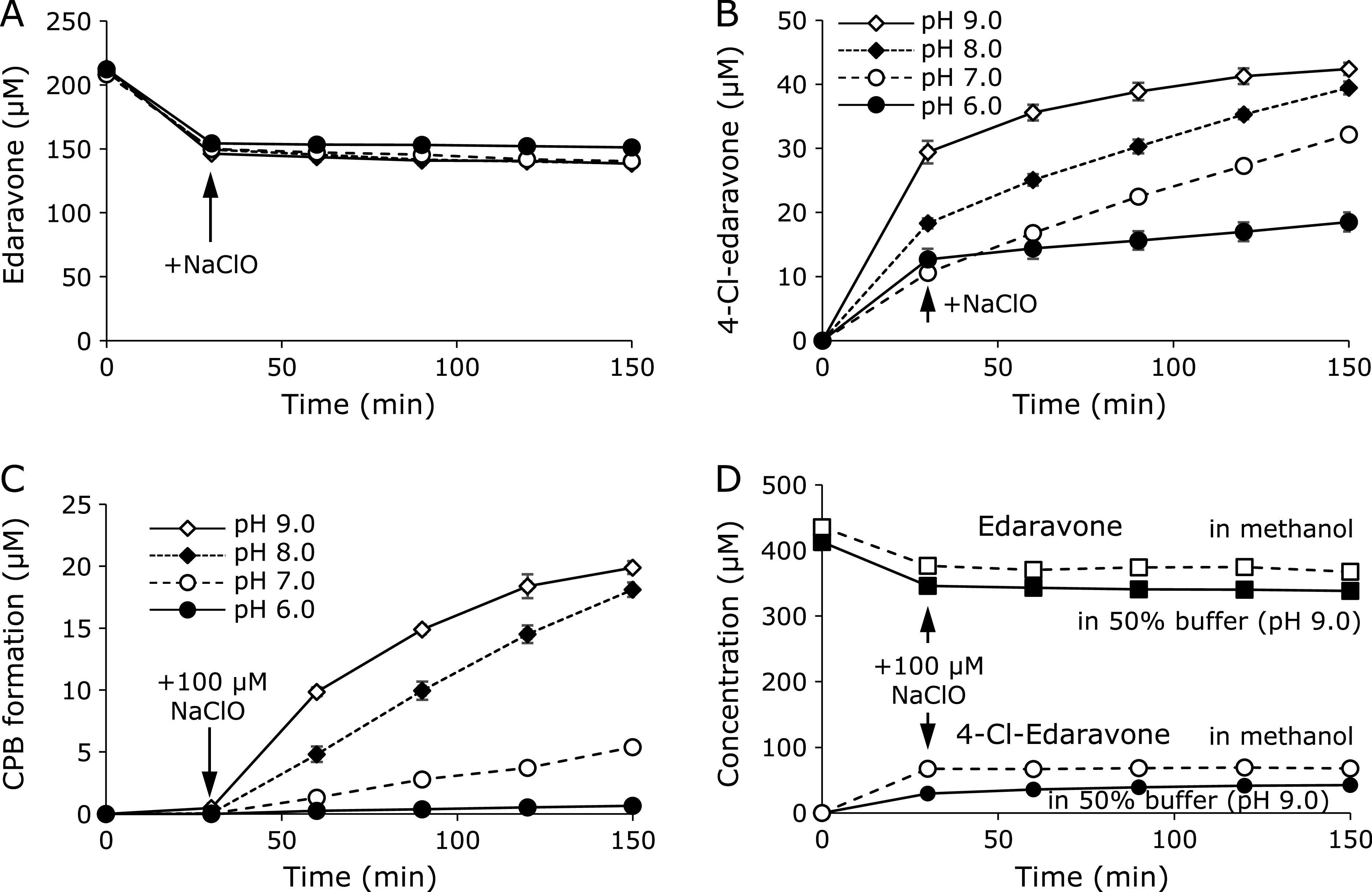
Edaravone oxidation by instantaneously added NaClO. (A) Edaravone (200 µM) oxidation by 100 µM NaClO addition at pH 6.0 (●), 7.0 (◯), 8.0 (◆), and 9.0 (◇). 4-Cl-Edaravone (B) and CPB (C) formation with reaction of 200 µM edaravone and 100 µM NaClO at pH 6.0 (●), 7.0 (◯), 8.0 (◆), and 9.0 (◇). (D) Edaravone degradation (squares) and 4-Cl-edaravone (circles) formation with an immediate addition of NaClO to edaravone in 50% buffer (pH 9.0) (closed symbols) or in methanol (open symbols).

Finally, to investigate the stoichiometry for the reaction of edaravone and ClO^−^, 100 µM NaClO was immediately added to 200 µM edaravone solution at pH 6.0, 7.0, 8.0 and 9.0. The concentration of edaravone was immediately reduced after NaClO addition, similar to 4-Cl-edaravone, and the decrement was approximately 70 µM at any pH (Fig. 5A), whereas 4-Cl-edaravone (Fig. 5B) and CPB (Fig. 5C) formations were dependent on pH. Table 3 shows the amounts of edaravone degradation and 4-Cl-edaravone and CPB formations in the instantaneous reaction of 200 µM edaravone and 100 µM ClO^−^ at various pHs. Edaravone degradation and CPB formation reached approximately 95 µM, equimolar with the added ClO^−^, at pH 9.0 where the reaction was the most efficient. Considering that the stoichiometric number for CPB production from the reaction of 4-Cl-edaravone and ClO^−^ was determined to be 1.0, the stoichiometric number for the reaction of edaravone and ClO^−^ is also estimated to be 1.0. These results indicate that one edaravone molecule reacts with one ClO^−^ to immediately form an unknown intermediate for 4-Cl-edaravone formation. However, interestingly, 4-Cl-edaravone was instantaneously formed after NaClO addition to edaravone methanolic solution whereas the formation was gradual in 50% buffer solution (pH 9.0), indicating that the reactions of water and ClO^−^ or edaravone were competing with the reaction of edaravone and ClO^−^ (Fig. 5D). The results suggested that the intermediate could be formed from edaravone, water and HOCl, which is produced from ClO^−^ and water.

**Fig. 6:**
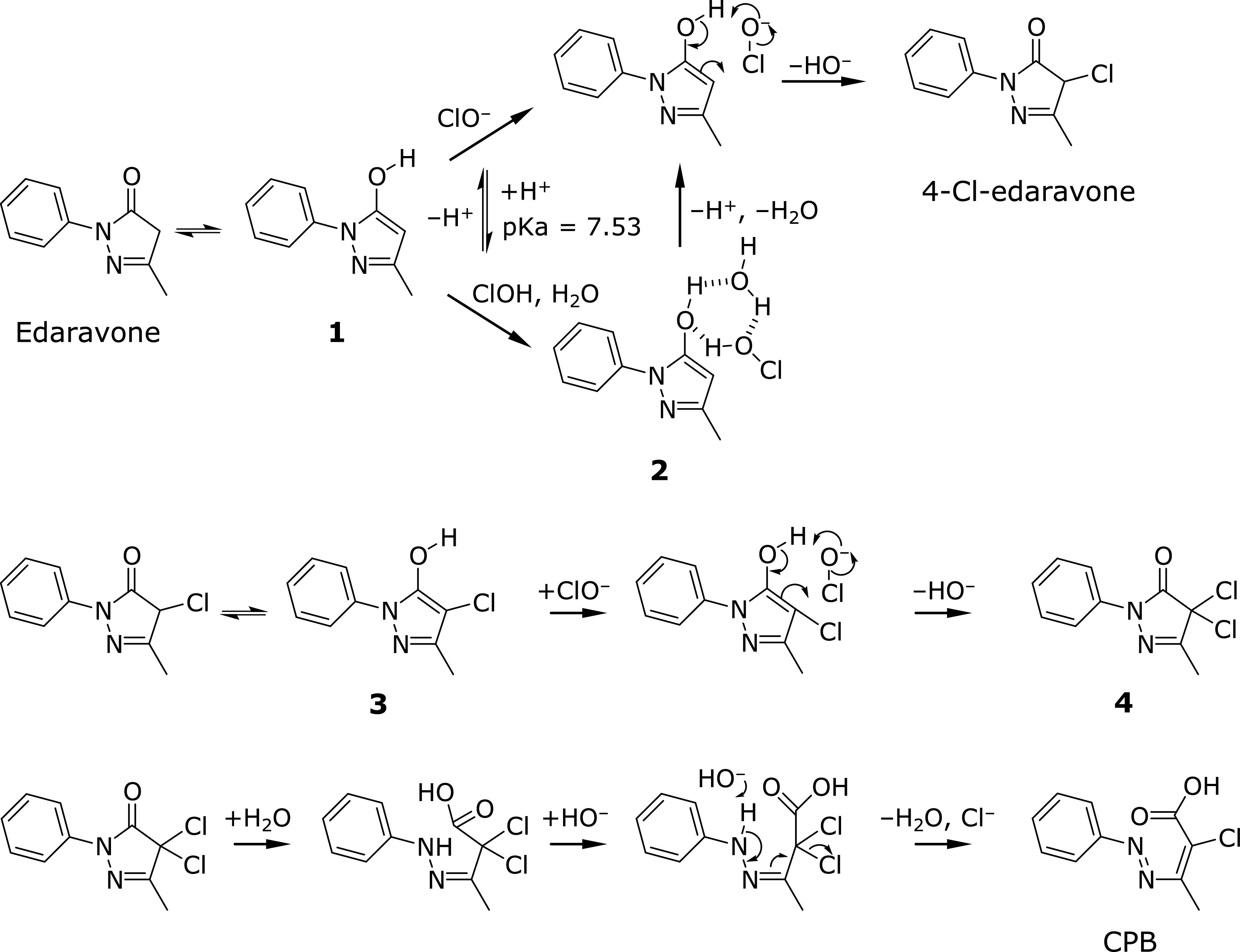
Plausible mechanisms for reactions of edaravone and 4-Cl-edaravone with ClO^−^.

From these results, we postulated the reaction mechanism for edaravone and ClO^−^ (Fig. 6). The enol form of edaravone (**1**) reacts with ClO^−^ or HOCl. When it reacts with ClO^−^, electron transfers rapidly occur to produce 4-Cl-edaravone. On the other hand, interaction of the enol of edaravone, HOCl, and H_2_O yields a stable cluster (**2**) as the intermediate with three hydrogen bonds between three OH groups. Deprotonation from the cluster molecule leads to 4-Cl-edaravone. Considering that the pKa value for HOCl is 7.53, it seems reasonable that 4-Cl-edaravone formation proceeded at pH above 8.0. The enol form of 4-Cl-edaravone (**3**) reacts with ClO^−^ to form 4,4-dichloro-edaravone (**4**) as an intermediate. The hydrolysis at the 1N-5C of 4,4-dichloro-edaravone and subsequent hydrogen absorption from its amino group and rearrangement of the 2N-3C double bond to C3-C4 occur to give CPB (Fig. 6). This process is thought to be dependent on hydrogen elimination which is caused by HO^−^. In fact, CPB production was greater under higher pH (Fig. 5C), supporting our hypothesis.

## Conclusion

We demonstrated that edaravone reacts with ClO^−^ at a comparable rate to thiols and sulfides, which are believed to be important endogenous ClO^−^ scavengers *in vivo*. These results indicate that edaravone is capable of reacting with ClO^−^ while co-existing with these antioxidants *in vivo*. The reaction yields 4-Cl-edaravone and CPB as primary and end products, respectively. From these results, edaravone treatment can be beneficial toward ClO^−^-related injuries such as inflammation, in which MPO is thought to be up-regulated. Furthermore, since 4-Cl-edaravone and CPB are ClO^−^-specific oxidation products, they can be good indicators for ClO^−^ formation *in vivo*. Therefore, if these compounds are detected from edaravone-treated patients with diseases such as brain infarction and ALS, it indicates that ClO^−^ production occurs in these pathological conditions.

## Figures and Tables

**Table 1:** Ratio of rate constants for edaravone and reference antioxidants [mean ± SD (*n* = 3)]

Reference	*k*_E_/*k*_R_	*k*_R_ (M^−1^s^−1^)
UA	24.6 ± 0.53	3.0 × 10^5^ ^a^
Met	1.21 ± 0.04	3.4 × 10^7^ ^b^
Cys	0.20 ± 0.01	3.6 × 10^8^ ^b^
GSH	0.46 ± 0.01	1.2 × 10^8^ ^b^

^a^see references (10)–(12), ^b^see reference (9).

**Table 2:** 4-Cl-edaravone degradation and CPB formation during reaction of 4-Cl-edaravone and sodium hypochlorite [µM, mean ± SD (*n* = 3)]

pH	Added NaClO	Δ4-Cl-edaravone	Added NaClO/Δ4-Cl-edaravone
6.0	100	94.2 ± 1.5	1.06 ± 0.02
7.0	50	47.8 ± 1.0	1.05 ± 0.02
	100	101.3 ± 3.8	0.99 ± 0.04
	200	194.1 ± 8.5	1.03 ± 0.05
8.0	100	97.7 ± 2.9	1.02 ± 0.03
9.0	100	98.9 ± 3.5	1.01 ± 0.03

**Table 3:** Edaravone degradation and 4-Cl-edaravone and CPB formation during reaction of edaravone and sodium hypochlorite [µM, mean ± SD (*n* = 3)]

pH	Added NaClO	ΔEdaravone	4-Cl-edaravone formation	CPB formation
6.0	100	60.9 ± 1.0	18.5 ± 1.5	0.65 ± 0.04
7.0	100	68.1 ± 2.9	32.2 ± 0.5	5.38 ± 0.30
8.0	100	71.6 ± 0.6	39.4 ± 0.1	18.1 ± 0.6
9.0	100	74.5 ± 4.3	42.4 ± 1.0	19.9 ± 0.5

## Abbreviations

ALS: amyotrophic lateral sclerosis
ClO^−^: hypochlorite
CPB: (*E*)-2-chloro-3-[(*E*)-phenyldiazenyl]-2-butenoic acid
Cys: cysteine
ECD: electrochemical detector
GSH: glutathione reduced form
Met: methionine
MPO: myeloperoxidase
OPB: 2-oxo-3-(phenylhydrazono)butanoic acid
TFA: trifluoroacetic acid
TOFMS: time-of-flight mass spectrometry
UA: uric acid
